# Numbers and narratives: how qualitative methods can strengthen the science of paediatric antimicrobial stewardship

**DOI:** 10.1093/jacamr/dlab195

**Published:** 2022-01-22

**Authors:** Charlotte Z. Woods-Hill, Anping Xie, John Lin, Heather A. Wolfe, Alex S. Plattner, Sara Malone, Kathleen Chiotos, Julia E. Szymczak

**Affiliations:** 1 Division of Critical Care Medicine, The Children’s Hospital of Philadelphia, 3401 Civic Center Blvd, Philadelphia, PA 19104, USA; 2 University of Pennsylvania Perelman School of Medicine, 3400 Civic Center Blvd, Philadelphia, PA 19104, USA; 3 The Leonard Davis Institute of Health Economics, University of Pennsylvania, 3641 Locust Walk # 210, Philadelphia, PA 19104, USA; 4 Department of Anesthesiology and Critical Care Medicine, Johns Hopkins University School of Medicine, 733 N Broadway, Baltimore, MD 21205, USA; 5 Armstrong Institute for Patient Safety and Quality, Johns Hopkins University School of Medicine, 750 E Pratt St., Baltimore, MD 21202, USA; 6 Division of Pediatric Critical Care Medicine, Department of Pediatrics, Washington University School of Medicine, 660 S Euclid Ave, St Louis, MO 63110, USA; 7 Division of Pediatric Infectious Disease, Department of Pediatrics, Washington University School of Medicine, 660 S Euclid Ave, St Louis, MO 63110, USA; 8 Department of Biostatistics, Epidemiology and Informatics, Perelman School of Medicine, University of Pennsylvania, 3400 Civic Center Blvd, Philadelphia, PA 19104, USA

## Abstract

Antimicrobial and diagnostic stewardship initiatives have become increasingly important in paediatric settings. The value of qualitative approaches to conduct stewardship work in paediatric patients is being increasingly recognized. This article seeks to provide an introduction to basic elements of qualitative study designs and provide an overview of how these methods have successfully been applied to both antimicrobial and diagnostic stewardship work in paediatric patients. A multidisciplinary team of experts in paediatric infectious diseases, paediatric critical care and qualitative methods has written a perspective piece introducing readers to qualitative stewardship work in children, intended as an overview to highlight the importance of such methods and as a starting point for further work. We describe key differences between qualitative and quantitative methods, and the potential benefits of qualitative approaches. We present examples of qualitative research in five discrete topic areas of high relevance for paediatric stewardship work: provider attitudes; provider prescribing behaviours; stewardship in low-resource settings; parents’ perspectives on stewardship; and stewardship work focusing on select high-risk patients. Finally, we explore the opportunities for multidisciplinary academic collaboration, incorporation of innovative scientific disciplines and young investigator growth through the use of qualitative research in paediatric stewardship. Qualitative approaches can bring rich insights and critically needed new information to antimicrobial and diagnostic stewardship efforts in children. Such methods are an important tool in the armamentarium against worsening antimicrobial resistance, and a major opportunity for investigators interested in moving the needle forward for stewardship in paediatric patients.

## Introduction

Antimicrobial stewardship (AS) is the coordinated effort to measure and improve how antimicrobials are prescribed by clinicians and used by patients in order to effectively treat infections, eliminate harms caused by unnecessary antibiotic use and combat antibiotic resistance.^1^ Antimicrobial resistance (AMR) is a growing threat with dire consequences for the world’s population: excess morbidity, mortality and cost.^2–6^ AS, including the growing subset of AS research focused on the concept of diagnostic stewardship, has been recognized as a key strategy to combat antibiotic resistance by optimizing antimicrobial selection and reducing antibiotic over use, but antibiotic overuse is still prevalent.^7,8^

Antibiotics are among the most commonly prescribed medications to both infants and children in the outpatient and inpatient setting. One-third of hospitalized children receive antibiotics for prophylactic use, many of which are broad-spectrum agents, and over half of children in the ICU receive antibiotics.^9–13^ While they can be life-saving, antibiotics can also cause significant harm to children, such as allergic reactions, *Clostridioides difficile*-associated diarrhoea, dangerous interactions with existing medications and renal injury; and overuse of antibiotics acts as a major driver of antibiotic resistance.^14–18^ While there have been notable reductions in antibiotic prescribing in children in the last few decades, up to 25%–50% of antibiotic prescriptions are inappropriate in dose, duration or drug choice, or not indicated.^10,19–22^

Combating AMR is an issue of global importance, considering both the harm that unnecessary antibiotics can cause patients and the complexities of the diagnostic and treatment decisions encountered in hospitalized children. Clinicians and researchers must use every tool possible to facilitate successful antimicrobial and diagnostic stewardship efforts in this population. This includes integrating an array of methodological approaches to generate knowledge about what works in stewardship, how and why. While the field has traditionally relied on quantitative methods to answer research questions, there is a growing recognition of the value of qualitative methods of inquiry. Qualitative methods generate different kinds of knowledge than that generated by quantitative methods.^23,24^ Through close examination of detailed narratives, observations of people interacting and cultural artifacts, researchers using qualitative methods strive to understand human behaviour and decision-making in context. When these methods are applied to research aimed at improving healthcare delivery, they can identify mechanisms underlying statistical correlations, inform the development of interventions and show how interventions work to produce observed outcomes.^25^

This article will describe the value of using qualitative methods to conduct research on antimicrobial and diagnostic stewardship in paediatrics. We will briefly review the key features of qualitative methods and describe the advantages of using them in stewardship research through the examination of qualitative studies on important topics in paediatric stewardship. We conclude by highlighting the role of qualitative methods in the multidisciplinary social and behavioural science approaches that are now recognized as fundamental to the success of antimicrobial and diagnostic stewardship.^26,27^ Young investigators seeking to make an impact in the science of paediatric stewardship can advance their research by embracing multiple approaches to knowledge generation and multidisciplinary collaborations.

## Key features of qualitative methods

Qualitative methods are used across academic disciplines (anthropology, sociology, psychology, communications) and for different purposes (basic research, applied and evaluation research, market research, user-centred design). Methods are informed by disciplinary norms, values and philosophies of knowledge.^28^ Researchers bring a set of assumptions about the basic entities that make up reality (ontology) and the nature of knowledge (epistemology) to the work of scholarly inquiry. There are different approaches to conducting qualitative research depending on discipline and objective. Common features, however, set it apart from the dominant approach to knowledge generation in biomedicine—quantitative methodology.^29^

Qualitative methods aim to generate knowledge about the world by understanding a phenomenon or topic in context and explore what or how something happens or is experienced by people through the systematic analysis of text or images.^30^ Qualitative research is oriented to understanding human experience and behaviour from the perspective of those who are the subject of study. Qualitative methods elicit information from people in an open-ended manner without imposing pre-determined measures. It stands in contrast to quantitative methods, which use standardized measurements deemed meaningful by the scientific community to obtain information about a phenomenon. Qualitative research uses interpretive approaches to data analysis, where patterns of meaning are identified and used to generate theory and hypotheses. Quantitative research uses statistical analysis of numerical data to test theories and hypotheses.^31^

Investigators should consider using qualitative methods in their work to investigate complex social and behavioural phenomena that are difficult to measure or count.^32^ Paediatric AS research has often used quantitative methods: for example, to assess the effectiveness of stewardship programmes in improving antibiotic use and patient outcomes.^33^ While this research is essential, it is insufficient to address many critical questions related to the design (e.g. what social, behavioural and contextual factors influence antibiotic prescribing); implementation (e.g. how an AS programme can be adapted to individual healthcare settings); and evaluation [e.g. why an AS programme is (not) effective in improving antibiotic prescribing practices] of AS interventions. Ultimately, stewardship is oriented towards changing the behaviour of people with different perspectives, motivations and constraints within complex organizations characterized by uncertainty.^34^ When researchers using qualitative methods they seek to ‘describe, decode, translate, and otherwise come to terms with the meaning, not the frequency, of certain more or less naturally occurring phenomena in the social world.’^35^ These methods are essential and powerful tools that can be used to answer critical research questions in stewardship (Table 1).

**Table 1.** Types of qualitative methods and examples of their use in stewardship

Qualitative method	Description	Examples of AS research questions answerable by the method	Impact/contribution
Semi-structured interview	Asking a research participant a series of pre-planned open-ended questions around a common idea or set of ideas to learn more about opinions, beliefs, and experiences, incorporating follow-up questions to generate more nuanced understanding.^112^	How do prescribers think about the role of the ASP team in the management of their patients? How do these perceptions influence willingness to accept ASP recommendations?	Allows for a more complex understanding of how people think about or make meaning around a certain topic. Interviews can also be useful for understanding more about a topic that might be considered sensitive.
Focus groups	Facilitated group discussion using a series of pre-planned, open-ended questions to stimulate conversation amongst participants. Groups usually consist of individuals with shared experience or other similarities.^113^	How does the practice culture of outpatient paediatrics influence the management of parent demand for antibiotics?	Like interviews, focus groups result in a deeper understanding of meaning and meaning-making amongst participants. Focus groups are used when the collective experiences of a group, and the interactions of group participants with each other, is of empirical interest.
Ethnography	Data are gathered by a trained observer who seeks to understand the social and cultural norms of group behaviour through observations of naturally occurring interaction.^114^	How do ASP teams approach other professions during hospital rounds?	Provides insight into influences on interactions that might not be articulated by research participants using other methods.
Thematic content analysis of documents or artifacts	Examining documents, imagery and other artifacts to understand how events unfold and how groups of people communicate social norms and express what matters.	How is responsibility communicated through a hospital’s protocols and policies towards antibiotic stewardship? What has changed over time? How?	Can help triangulate information when used in conjunction with other sources. Provides information about what is occurring in an environment, helps with historical knowledge, or helps with background information.

ASP, AS programme.

Perhaps the most powerful approach for investigators in paediatric stewardship, who may have more familiarity with quantitative methods, is to consider employing a mixed methods approach to research. The integration of qualitative and quantitative methods is growing in popularity in the health sciences and is increasingly accepted as a way to achieve both breadth and depth of inquiry.^36–38^ The ‘mix’ of quantitative and qualitative approaches can maximize the strengths and minimize the weaknesses of each approach, increase the validity of research results and contribute to theory development.^39^ There are several specific advantages to mixed methods studies. For example, they can help researchers to illustrate and then understand contradictions between quantitative results and qualitative findings. They have the potential to facilitate research that is informed not only by the interests of the scientific community, but also the people who have lived experience with the topic of study. The methodological flexibility inherent in mixed methods designs can allow for multiple questions to be answered within one study. Ultimately, mixed methods designs can generate richer and more comprehensive data than qualitative or quantitative designs alone.^40^

Mixed methods approaches can be complex to achieve in practice, and require careful attention to timing, weighting and the nature of interface between the methods.^41^ Table 2 shows examples of AS research using different mixed methods designs.^42^

**Table 2.** Types of mixed methods studies and examples in AS research

Core mixed methods design	Description	Example in AS research
Convergent design or concurrent design	Concurrent or parallel collection of qualitative and quantitative data with mixing of results after analysis but during interpretation of the findings	Quantitative survey data and qualitative interview data were collected and triangulated to evaluate the implementation of AS programmes in Missouri hospitals.^115^
Explanatory sequential design	Initial collection and analysis of quantitative data that informs the subsequent collection, analysis and interpretation of qualitative data	A cluster-randomized control trial was conducted to quantitatively assess the effect of an outpatient AS intervention on broad-spectrum antibiotic prescribing by primary care paediatricians,^116^ which was followed by a qualitative interview study to explore the perceptions of participating paediatricians regarding the outpatient AS intervention.^43^
Exploratory sequential design	Initial collection, analysis and interpretation of qualitative data that is used to inform the collection of quantitative data	Qualitative data on factors influencing antibiotic prescribing were collected by semi-structured interviews, which informed the design of instruments used in a discrete choice experiment to quantify factors influencing antibiotic prescribing.^117^

## Qualitative research on key issues in paediatric AS

### Paediatric clinician attitudes toward AS

Qualitative methods such as interviews and focus groups have been used to better understand paediatric clinician attitudes towards antibiotic use and AMR in both the inpatient and outpatient settings. Several studies have identified insight to inform future stewardship work: the role of parental pressure in prescribing antibiotics, clinician mistrust and skepticism towards stewardship guidelines, clinician concerns about medico-legal difficulties if failing to prescribe antibiotics, and clinician dissatisfaction with loss of autonomy from restrictions placed on their decision-making by stewardship interventions.^43–46^ Collectively, these findings highlight the value of building clinician trust prior to implementing interventions, and the importance in educating both prescribers and parents during AS efforts.

### Identifying influences on paediatric clinicians’ antimicrobial prescribing behaviours

The Antibiotic Mapping of Prescribing (ABMAP) study used multiple methods to understand the barriers to judicious antibiotic prescribing.^47^ Three methods (survey, focus groups/interviews, follow-up survey) were used to gather information from paediatric clinicians in the UK’s Birmingham Children’s Hospital. The initial survey identified that the most significant barriers to appropriate antibiotic prescribing were pressure from patient families or senior colleagues, issues related to the laboratory such as delayed results, and lack of appropriate antibiotic-related training. During the subsequent focus groups and interviews, clinicians expressed concern for AMR but felt that individual patient treatment does not significantly impact the societal risk of AMR. Additionally, most clinicians expressed desire for mechanisms to be put in place to aid in changing prescribing practices. The final phase consisted of an online survey to elicit possible solutions to change prescribing behaviours. Participating clinicians had a strong preference for direct instruction rather than being provided data to guide decision-making and preferred instruction come from subject experts over electronic tools.

### Paediatric AS in low-resource settings

AS efforts in low-resource settings are of critical importance in combating the global burden of AMR given that the prevalence of antibiotic-resistant pathogens is inversely proportion to a country’s gross national income.^48^ What may appear to be a local problem in fact has wide-ranging consequences: increasing antimicrobial utilization, high rates of self-medication with antimicrobials, extensive use of antimicrobials to prevent and treat diseases in animals, lack of hand hygiene and high frequency of travelling between countries in low-resource settings all contribute significantly to the global rise in AMR.^49^ While a detailed exploration of the myriad challenges of AS work in such settings is beyond the scope of this paper, we will highlight an example of qualitative stewardship efforts undertaken to date, which offers both important initial insights and directions for future study.

In response to previous studies raising concern for over-prescription of antibiotics in Tanzania, a 2021 qualitative study used in-depth interviews to characterize clinician experiences prescribing antibiotics to children less than 5 years of age.^50^ Prescribers reported that antibiotics are often prescribed solely on physical exam and history, and laboratory testing is rarely available. Prescribers often rely on previous experiences; however, published guidelines, such as those from WHO, were identified as useful tools for decision-making. Challenges in reconciling maternal expectations for antibiotics with maternal tendencies to alter dosing schedules or discontinue antibiotics early was identified as a significant barrier to appropriate antibiotic use. Among low-income families, additional challenges include purchasing antibiotics directly from the pharmacy without being first seen in a healthcare facility, buying only a portion of an antibiotic course, or buying a more affordable antibiotic instead of what is considered the best treatment option.

Participating prescribers also identified other healthcare actors, including pharmacies and private facilities, as additional obstacles for AS: citing the quality of drugs, difficulty with drug availability, sales of antibiotics without a prescription and profit-driven practices as all potentially contributing to antibiotic misuse. However, prescribers did not feel that AMR was a problem in their daily practice. Overall, they perceived AMR as a consequence for individual patients misusing antibiotics rather than a public health concern.

From these insights into prescriber experiences, the investigators outlined specific interventions to improve prescribing practices. For example, trusted guidelines were updated to specifically address clinical situations in which symptomatic patients do not need antibiotics, and the public health impact of AMR was emphasized as a societal problem.

### Parents’ perspectives on antibiotics and antibiotic stewardship in children

A very commonly reported barrier to judicious prescribing in paediatrics is parent or caregiver pressure for unnecessary antibiotics. Consequently, multiple qualitative studies have been conducted to understand how parents perceive the use of antibiotics in the care of their children. Most qualitative research studies on this topic have focused on the outpatient setting, specifically around acute respiratory infections (ARTI) and acute otitis media. For years, the perception by clinicians was that parental demand for antibiotics contributes to overuse.^42^ Recent qualitative research suggests that paediatricians may overestimate parent demand for antibiotics, and this perception may be driving paediatrician overprescribing.^51^ While parents have misconceptions about the role of antibiotics in acute lower respiratory infections, recent evidence shows that parents are becoming more sophisticated in their knowledge about when antibiotics are necessary.^52,53^ For example, Coxeter *et al*.^54^ performed 401 phone interviews regarding antibiotic use for common respiratory infections. While parents overestimated the benefit of antibiotics on reducing symptom duration and believed that antibiotics lead to reduced rates of complications, they did recognize that antibiotics can cause harm. Further, in a study by Halls and colleagues,^55^ the parental expectations for antibiotic prescriptions were differential based on perceptions of risk, with antibiotics felt to be necessary in younger and severely ill children to a greater extent.

Szymczak *et al*.^56^ performed semi-structured interviews with parents before their appointment with a paediatrician to understand their knowledge and expectations about antibiotic use for an ARTI. They found that parents have a sense of wariness when child is prescribed antibiotics, perceive that antibiotic overuse is a problem that is driven by the demands of other parents, and have preference for alternative treatments. Despite these beliefs, parents did not have concerns about antibiotic resistance development due to over-prescription of antibiotics for ARTI, and most hesitation about the use of antibiotics pertained to the side effects of the medications themselves. Van Hecke *et al*.^57^ performed semi-structured interviews with parents of children who were treated for a recent ARTI. Parents considered their families to be at low risk for developing AMR since they infrequently used antibiotics, and few families had concerns about the development of antibiotic resistance. Similarly, they did not perceive that their families’ use of antibiotics contributed to the development of AMR at the societal level.

Only two studies have explored antibiotic prescribing and AMR beliefs among hospitalized paediatric populations. Warembourg *et al*.^58^ performed telephone interviews with 63 families after discharge from hospital regarding adherence to antibiotic usage after discharge. Sixty percent of families were non-adherent to antibiotic use and misunderstood the importance of taking antibiotics. Diorio *et al*.^59^ interviewed parents of children, children and healthcare workers in a patient population that was either receiving chemotherapy or haematopoietic stem cell transplant for cancer. The chance of death and infection were important drivers in antibiotic prophylaxis for families, patients and healthcare workers. All participants recognized that AMR was an important issue, however, it did not have an impact on individual decision-making about infection prophylaxis. Resistance was felt to be a community issue and was valued differently than the individual needs of a single patient.

Additional research about parental perspectives on antibiotic prescribing in various inpatient populations would optimize inpatient stewardship interventions. In particular, future studies exploring the origins and implications of the perception that sicker or vulnerable children require antibiotics would be beneficial. This perception has been identified in both the outpatient ARTI and inpatient oncology context and may persist across a diverse spectrum of inpatient care areas.

## Qualitative research on paediatric stewardship in particular clinical contexts: ICUs, oncology and congenital heart disease

### Qualitative stewardship research in the paediatric ICU

Over 50% of patients in the paediatric ICU (PICU) receive antibiotics, which are typically parenteral and many times are combination regimens of multiple agents.^9^ While a significant proportion of this antibiotic use, of course, reflects the high proportion of PICU patients who are ill with either confirmed or suspected bacterial infections, many PICU patients likely receive antibiotics in the absence of infection.^60–62^ Infectious and non-infectious aetiologies of shock and multi-organ failure in critically ill children often have similar presenting symptoms, and, in the absence of confirmatory culture data, no single symptom, test or biomarker can reliably distinguish the two.^63–66^ Compounding this diagnostic challenge, national guidelines and national collaboratives call for rapid treatment of suspected sepsis in children with broad-spectrum antibiotics, despite little data supporting this practice in patients without shock.^67,68^ Simultaneously, evidence of harm from antibiotic overuse in paediatric patients continues to grow.^69–71^ PICU clinicians are thus faced with competing pressures to ensure rapid antibiotic administration to critically ill patients with sepsis, while avoiding antibiotic overuse in uninfected patients—a situation made particularly challenging given the diagnostic uncertainty that results from sometimes vague symptoms of sepsis.

This makes the PICU a challenging, but particularly important, environment for AS work.^72^ Such work is being done, but data on the impact of AS programmes in the PICU setting are somewhat limited compared with impact on non-PICU paediatric patients.^33^ In the PICU in particular, diagnostic stewardship work therefore offers an important complementary strategy that may facilitate optimal use of antibiotics by working one step upstream in the diagnosis-treatment cascade. Qualitative methods have been used to advance diagnostic stewardship in the PICU setting with regard to blood culture practices. Blood cultures are fundamental in the diagnosis and treatment of bacteraemia, a primary cause of sepsis and associated morbidity and mortality in PICU patients.^60^ However, blood cultures can be used excessively in PICU patients when the pre-test probability of bacteraemia is low, increasing the chance of obtaining a false positive result.^73–76^ False positive blood cultures cause patient harm and strain on healthcare resources: repeat testing, unnecessary antibiotics, longer length of stay, exposure to additional procedures and consultations, and increased cost.^77,78^ Within the PICU setting, growing evidence suggests blood cultures can be safely reduced using a diagnostic stewardship approach, and qualitative methods have been a critical part of establishing this evidence.^79,80^ A modified Delphi approach was used to develop recent national consensus recommendations that describe clinical scenarios appropriate for targeted blood culture reduction.^81^ In addition, part of the work that preceded development of those guidelines was qualitative exploration of blood culture ordering practices by PICU clinicians using semi-structured interviews and qualitative content analysis.^82^ Reflexive practices (e.g. always ordering a blood culture from a patient with fever and a central venous catheter), the local unit culture and fear of missing sepsis emerged in that work as potential drivers of blood culture overuse, and were confirmed in subsequent surveys of multiple sites.^82,83^ Targeting these factors may be important to further reduce excess blood cultures and promote broad implementation of this diagnostic stewardship approach.

### Paediatric oncology patients

Children with malignancies undergoing chemotherapy have a high risk of morbidity and mortality from infections, and thus present unique challenges to AS efforts.^84–86^ However, the negative consequences from things like excessive entry into central venous catheters for frequent blood cultures, adverse effects of unnecessary antibiotics such as kidney injury and AMR are significant, and justify efforts to include such patients in stewardship work.^86^ Qualitative methods can offer important insights into how to best accomplish this; for example, parent/caregiver perceptions of early discharge during febrile neutropenia via semi-structured interviews, meta-ethnography and focus groups suggested it was a safe option under certain circumstances.^87–89^ Another example is a recent consensus conference that employed Delphi methods to develop two recommendations focused on blood culture reduction for immunocompromised PICU patients.^81^ Efforts to include paediatric oncology patients in diagnostic/antimicrobial stewardship initiatives and research remain important, and qualitative approaches offer opportunities to more richly characterize how this can be successful.

### Paediatric cardiac surgical patients

Children with congenital heart disease hospitalized in the cardiac ICU also present unique challenges related to AS, including increased risk of infection given frequent use of invasive devices and possible comorbid immune system abnormalities (e.g. asplenia in cases of heterotaxy, T cell dysfunction in patients with DiGeorge syndrome), as well as the possibility of experiencing higher severity of illness in the setting of infection in the setting of complex cardiopulmonary physiology.^90^ Higher mortality rates are seen for nosocomial infections in the perioperative period in these patients, with the use of extracorporeal membrane oxygenation being an independent risk factor for infection.^91^ As with paediatric oncology patients, qualitative research on antimicrobial or diagnostic stewardship in children in the cardiac ICU setting is limited. However, paediatric cardiac ICU patients have been successfully included in collaborative qualitative work to reduce unnecessary use of blood cultures; and predictors of disagreement with AS programme recommendations to modify antibiotic therapy in this population have been examined using qualitative methods.^81,92,93^ There is a precedent for using qualitative methods in stewardship research on the care of paediatric cardiac ICU patients, with further study needed.

## The use of qualitative methods in multidisciplinary stewardship research: opportunities for collaboration

Given the urgent need to improve the impact of stewardship and sustain its effects, there are many opportunities for paediatric clinical investigators to engage in multidisciplinary collaborative research efforts. Antibiotic and diagnostic stewardship efforts are increasingly being informed by disciplines from the behavioural, social, management and engineering disciplines that have not, until relatively recently, been integrated into clinical medicine. Traditional clinical research has focused on establishing *clinical evidence* (i.e. what are the most effective tests, therapies and overall care for a given clinical condition?). Increasingly researchers are also shifting their attention to the equally important domains of *practice change*, *decision-making* and *the impact of care delivery on outcomes and value*.^94^ Key disciplines emerging as tools for addressing these domains include implementation science, human factors engineering, quality improvement methods, change leadership/management principles, patient-centred outcomes research methods and healthcare economics. These disciplines, which integrate both qualitative and quantitative methods of inquiry, can contribute to the science of stewardship in paediatrics. Engagement in multidisciplinary collaborative research can equip an investigator with an expanded skillset and opportunities for academic development.

Implementation science is increasingly being applied to research in stewardship.^95^ Implementation science is the scientific study of methods to promote the uptake of evidence-based clinical treatments, practices and interventions into routine use.^96^ Implementation science studies often incorporate qualitative and mixed-methods elements into their design, and benefit considerably from the rich data acquired from these methods, especially about the impact of context on implementation.^97^ The importance of implementation science to translate clinical evidence into real practice change in both adult and paediatric conditions has been increasingly recognized.^98–100^ Funding bodies are now prioritizing implementation science proposals, including for those investigators in the career-development phase, representing an emerging path for paediatric clinician-scientists interested in innovative approaches to stewardship efforts.^101,102^

Also crucial to the goal of changing behaviour in healthcare delivery is greater understanding of the role that work systems and environments play in influencing decision-making. As a discipline, human factors and ergonomics (HFE) focuses on how people interact with their environment. By improving the design of the work environment and tools that people use to accomplish their tasks, human factors science aims to enhance safety, efficiency and efficacy. HFE is increasingly applied to healthcare, in which it strives to achieve two primary goals: to support the healthcare professionals in both their cognitive and physical work and to promote high quality and safe care for patients.^103^ This scientific field has typically been leveraged to better understand causes of medical errors and to improve patient safety, the ‘Swiss Cheese Model’ being one of the most familiar concepts in understanding how a medical error is triggered and the gaps in the system that allow it to reach the patient.^104^ However, HFE can also be leveraged for paediatric stewardship work, including new models of HFE have been developed that incorporate the central role of the patient and his/her family.^105^

The application of social psychological and behavioural economic theories to medical decision-making, including antibiotic prescribing, has grown in recent years.^106^ Daniel Kahneman’s oft-cited cognitive model describing system 1 and system 2 modes of thinking provides a useful example.^107^ System 1 processes are faster and often times automatic; they are governed by intuition, experience and emotion or affect. System 2 processes are analytical, deliberative and logical, and are thus slower. In the example of paediatric diagnostic stewardship, a paediatric resident on call for the first time in a highly resourced PICU on a busy night might order a blood culture because of a combination of inexperience or worry about the potential of missing a chance to diagnose bacteraemia (system 1) but if given enough time to consider the diagnostic yield of the blood culture, the clinical likelihood of bacteraemia and the time to discuss the question with a senior clinician, they might decide to continue clinical observation without ordering a blood culture (system 2). Behavioural economics approaches to intervention design, through the application of ‘nudges’ to subtly change the choice architecture of clinical settings, have increasingly been used in AS.^106,108^

Questions remain about how to best address the core domains of clinical evidence, practice change and decision-making to optimize patient outcomes and in antimicrobial and diagnostic stewardship. Combining knowledge and understanding from diverse disciplines such as implementation science, human factors and ergonomics, behavioural economics, and epidemiology with evidence from basic science and clinical research forms the foundation of translational research, defined by the NIH as: ‘applying basic science and clinical research findings to more rapid and timely adoption of best practices to the bedside while optimizing cost-effectiveness and ultimately healthcare value of our diagnostic and therapeutic modalities.’^109^ We encourage paediatric clinicians and researchers to expand their methodological toolkit to incorporate qualitative and mixed methods approaches and collaboration with other disciplines in order to meaningfully, and sustainably, move the needle for stewardship in children.

## Limitations of qualitative methods

While qualitative methods offer considerable benefits for stewardship research, as with any method, they have limitations. Qualitative methods do not produce results that are statistically generalizable. The interpretive nature of qualitative findings are not easily reproduced.^110^ Each particular qualitative method also has its own specific limitations, such as the time-consuming nature of ethnography and observations, the challenges of navigating group dynamics such that all voices are heard during focus groups, and the risk of exaggerated or untruthful responses to sensitive questions during interviews.^111^ We encourage researchers to examine both the overall strengths and limitations of a qualitative approach they are considering using. The value of the method for answering the research question should be balanced with its specific weaknesses, and strategies to mitigate these weaknesses identified in advance.

## Conclusions

Combating the growing threat of AMR will require focused efforts in both adults and children, and a conscious decision to embrace a variety of research methods to make significant progress. We believe strongly that this includes qualitative methods, which can offer a rich understanding of the contextual and human elements that impact diagnostic and treatment decisions but may escape capture with quantitative methods. Paediatric patients will benefit from work that recognizes this important truth, and paediatric researchers who adopt this perspective have a wealth of opportunities at their fingertips to conduct rigorous research that will contribute to our ability to mitigate AMR and help patients across the globe.

## References

[1] CDC . Core Elements of Antibiotic Stewardship. https://www.cdc.gov/antibiotic-use/core-elements/.

[2] Klevens RM , Edwards JR, Richards CL Jr et al Estimating health care-associated infections and deaths in U.S. hospitals, 2002. Public Health Rep 2007; 122: 160–6. 17357358 10.1177/003335490712200205 PMC1820440

[3] Roberts RR , Hota B, Ahmad I et al Hospital and societal costs of antimicrobial-resistant infections in a Chicago teaching hospital: implications for antibiotic stewardship. Clin Infect Dis 2009; 49: 1175–84. 19739972 10.1086/605630

[4] Mauldin PD , Salgado CD, Hansen IS et al Attributable hospital cost and length of stay associated with health care-associated infections caused by antibiotic-resistant Gram-negative bacteria. Antimicrob Agents Chemother 2010; 54: 109–15. 19841152 10.1128/AAC.01041-09 PMC2798544

[5] Filice GA , Nyman JA, Lexau C et al Excess costs and utilization associated with methicillin resistance for patients with *Staphylococcus aureus* infection. Infect Control Hosp Epidemiol 2010; 31: 365–73. 20184420 10.1086/651094

[6] Goff DA , Kullar R, Goldstein EJC et al A global call from five countries to collaborate in antibiotic stewardship: united we succeed, divided we might Fail. Lancet Infect Dis 2017; 17: e56–63. 27866945 10.1016/S1473-3099(16)30386-3

[7] CDC . Antibiotic Resistance Threats in theUnited States, 2019. https://www.cdc.gov/drugresistance/pdf/threats-report/2019-ar-threats-report-508.pdf.

[8] WHO . Diagnostic Stewardship: a Guide to Implementation in Antimicrobial Resistance Surveillance Sites. 2016. https://apps.who.int/iris/handle/10665/251553.

[9] Versporten A , Sharland M, Bielicki J et al The antibiotic resistance and prescribing in European Children project: a neonatal and pediatric antimicrobial web-based point prevalence survey in 73 hospitals worldwide. Pediatr Infect Dis J 2013; 32: e242–53. 23838740 10.1097/INF.0b013e318286c612

[10] Tribble AC , Lee BR, Flett KB et al Appropriateness of antibiotic prescribing in United States children’s hospitals: a national point prevalence survey. Clin Infect Dis 2020; 71: e226–34. 31942952 10.1093/cid/ciaa036

[11] Chai G , Governale L, McMahon AW et al Trends of outpatient prescription drug utilization in US children, 2002–2010. Pediatrics 2012; 130: 23–31. 22711728 10.1542/peds.2011-2879

[12] Feudtner C , Dai D, Faerber J et al Pragmatic estimates of the proportion of pediatric inpatients exposed to specific medications in the USA. Pharmacoepidemiol Drug Saf 2013; 22: 890–8. 23704075 10.1002/pds.3456 PMC3810715

[13] Hufnagel M , Versporten A, Bielicki J et al High rates of prescribing antimicrobials for prophylaxis in children and neonates: results from the antibiotic resistance and prescribing in European Children Point Prevalence Survey. J Pediatric Infect Dis 2019; 8: 143–51. 10.1093/jpids/piy019 29579259

[14] Spellberg B , Blaser M, Guidos RJ et al Combating antimicrobial resistance: policy recommendations to save lives. Clin Infect Dis 2011; 52 Suppl 5: S397–428. 21474585 10.1093/cid/cir153 PMC3738230

[15] Shehab N , Lovegrove MC, Geller AI et al US emergency department visits for outpatient adverse drug events, 2013–2014. JAMA 2016; 316: 2115–25. 27893129 10.1001/jama.2016.16201 PMC6490178

[16] Gerber JS , Ross RK, Bryan M et al Association of broad- vs narrow-spectrum antibiotics with treatment failure, adverse events, and quality of life in children with acute respiratory tract infections. JAMA 2017; 318: 2325–36. 29260224 10.1001/jama.2017.18715 PMC5820700

[17] Bolhuis MS , Panday PN, Pranger AD et al Pharmacokinetic drug interactions of antimicrobial drugs: a systematic review on oxazolidinones, rifamycines, macrolides, fluoroquinolones, and β-lactams. Pharmaceutics 2011; 3: 865–913. 24309312 10.3390/pharmaceutics3040865 PMC3857062

[18] Tamma PD , Turnbull AE, Harris AD et al Less is more: combination antibiotic therapy for the treatment of gram-negative bacteremia in pediatric patients. JAMA Pediatr 2013; 167: 903–10. 23921724 10.1001/jamapediatrics.2013.196 PMC6857628

[19] Vaz LE , Kleinman KP, Raebel MA et al Recent trends in outpatient antibiotic use in children. Pediatrics 2014; 133: 375–85. 24488744 10.1542/peds.2013-2903 PMC3934343

[20] Klatte JM . Pediatric antimicrobial stewardship programs: current perspectives. Pediatric Health Med Ther 2020; 11: 245–55. 32801990 10.2147/PHMT.S224774 PMC7383043

[21] Fleming-Dutra KE , Hersh AL, Shapiro DJ et al Prevalence of inappropriate antibiotic prescriptions among US ambulatory care visits, 2010–2011. JAMA 2016; 315: 1864–73. 27139059 10.1001/jama.2016.4151

[22] Godbout EJ , Pakyz AL, Markley JD et al Pediatric antimicrobial stewardship: state of the art. Curr Infect Dis Rep 2018; 20: 39. 30069834 10.1007/s11908-018-0644-7

[23] Carter SM , Little M. Justifying knowledge, justifying method, taking action: epistemologies, methodologies and methods in qualitative research. Qual Health Res 2007; 17: 1316–28. 18000071 10.1177/1049732307306927

[24] Maxwell JA . Qualitative Research Design: an Interactive Approach. 3rd edn. Sage, 2013.

[25] Forman J , Creswell JW, Damschroder L et al Qualitative research methods: key features and insights gained from use in infection prevention research. Am J Infect Control 2008; 36: 764–71. 18834752 10.1016/j.ajic.2008.03.010

[26] Donisi V , Sibani M, Carrara E et al Emotional, cognitive and social factors of antimicrobial prescribing: can antimicrobial stewardship intervention be effective without addressing psycho-social factors? J Antimicrob Chemother 2019; 74: 2844–7. 31299072 10.1093/jac/dkz308

[27] Lorencatto F , Charani E, Sevdalis N et al Driving sustainable change in antimicrobial prescribing practice: how can social and behavioural sciences help? J Antimicrob Chemother 2018; 73: 2613–24. 30020464 10.1093/jac/dky222

[28] Guba EG , Lincoln YS. Competing paradigms in qualitative research. In: Denzin NK, Lincoln YS, eds. Handbook of Qualitative Research. SAGE Publications, 1994; 105–17.

[29] Rendle KA , Abramson CM, Garrett SB et al Beyond exploratory: a tailored framework for designing and assessing qualitative health research. BMJ Open 2019; 9: e030123. 10.1136/bmjopen-2019-030123 PMC6720470 31462482

[30] Hays D , Singh AA. Qualitative Inquiry in Clinical and Educational Settings. The Guilford Press, 2012.

[31] Duffy ME . Designing nursing research the qualitative-quantitative debate. J Adv Nurs 1985; 10: 225–32. 3848448 10.1111/j.1365-2648.1985.tb00516.x

[32] Curry LA , Nembhard IM, Bradley EH. Qualitative and mixed methods provide unique contributions to outcomes research. Circulation 2009; 119: 1442–52. 19289649 10.1161/CIRCULATIONAHA.107.742775

[33] Araujo da Silva AR , Albernaz de Almeida Dias DC, Marques AF et al Role of antimicrobial stewardship programmes in children: a systematic review. J Hosp Infect 2018; 99: 117–23. 28807835 10.1016/j.jhin.2017.08.003

[34] Szymczak JE , Newland J. The social determinants of antimicrobial prescribing: implications for stewardship. In: Barlam TF, Neuhauser MM, Trivedi KK et al, eds. Society for Healthcare Epidemiology of America: Practical Implementation of an Antimicrobial Stewardship Program. Cambridge University Press, 2018.

[35] Van Maanen J . Reclaiming qualitative methods for organizational research: a preface. Adm Sci Q 1979; 24: 520–6.

[36] Carr LT . The strengths and weaknesses of quantitative and qualitative research: what method for nursing? J Adv Nurs 1994; 20: 716–21. 7822608 10.1046/j.1365-2648.1994.20040716.x

[37] Choy LT . The strengths and weaknesses of research methodology: comparison and complimentary between qualitative and quantitative approaches. IOSR-JHSS 2014; 19: 99–104.

[38] Johnson RB , Onwuegbuzie AJ, Turner LA. Toward a definition of mixed methods research. J Mix Methods Res 2007; 1: 112–33.

[39] Morse JM . Approaches to qualitative and quantitative methodological triangulation. Nurs Res 1991; 40: 120–3. 2003072

[40] Mixed Methods: Integrating Quantitative and Qualitative Data Collection and Analysis While Studying Patient-Centered Medical Home Models, AHRQ Publication No: 13-0028-EF. 2013. http://www.ahrq.gov.

[41] Morse JM , Niehaus L. Mixed Method Design: Principles and Procedures. Left Coast Press, 2009.

[42] Creswell JW , Plano Clark VL. Designing and Conducting Mixed Methods Research. SAGE Publications, 2017.

[43] Szymczak JE , Feemster KA, Zaoutis TE et al Pediatrician perceptions of an outpatient antimicrobial stewardship intervention. Infect Control Hosp Epidemiol 2014; 35 Suppl 3: S69–78. 25222901 10.1086/677826

[44] Mauffrey V , Kivits J, Pulcini C et al Perception of acceptable antibiotic stewardship strategies in outpatient settings. Med Mal Infect 2016; 46: 285–93. 27475666 10.1016/j.medmal.2016.06.006

[45] Zetts RM , Stoesz A, Garcia AM et al Primary care physicians’ attitudes and perceptions towards antibiotic resistance and outpatient antibiotic stewardship in the USA: a qualitative study. BMJ Open 2020; 14: e034983. 10.1136/bmjopen-2019-034983 PMC7365421 32665343

[46] Stach LM , Hedican EB, Herigon JC et al Clinicians’ attitudes towards an antimicrobial stewardship program at a children’s hospital. J Pediatric Infect Dis Soc 2012; 1: 190–7. 26619407 10.1093/jpids/pis045

[47] Bashir A , Gray J, Bashir S et al Critical points in the pathway of antibiotic prescribing in a children’s hospital: the Antibiotic Mapping of Prescribing (ABMAP) study. J Hosp Infect 2019; 101: 461–6. 30071268 10.1016/j.jhin.2018.07.038

[48] Savoldi A , Carrara E, Gladstone BP et al Gross national income and antibiotic resistance in invasive isolates: analysis of the top-ranked antibiotic-resistant bacteria on the 2017 WHO priority list. J Antimicrob Chemother 2019; 74: 3619–25. 31730162 10.1093/jac/dkz381

[49] Godman B , Egwuenu A, Haque M et al Strategies to improve antimicrobial utilization with a special focus on developing countries. Life 2021; 11: 528. 34200116 10.3390/life11060528 PMC8229985

[50] Emgård M , Mwangi R, Mayo C et al Tanzanian primary healthcare workers’ experiences of antibiotic prescription and understanding of antibiotic resistance in common childhood infections: a qualitative phenomenographic study. Antimicrob Resist Infect Control 2021; 10: 94. 34176486 10.1186/s13756-021-00952-5 PMC8237496

[51] Finkelstein JA , Dutta-Linn M, Meyer R et al Childhood infections, antibiotics, and resistance. Clin Pediatr (Phila) 2014; 53: 145–50. 24137024 10.1177/0009922813505902 PMC4089954

[52] Mangione-Smith R , McGlynn EA, Elliott MN et al The relationship between perceived parental expectations and pediatrician antimicrobial prescribing behavior. Pediatrics 1999; 103: 711–8. 10103291 10.1542/peds.103.4.711

[53] Vaz LE , Kleinman KP, Lakoma MD et al Prevalence of parental misconceptions about antibiotic use. Pediatrics 2015; 136: 221–31. 26195539 10.1542/peds.2015-0883 PMC4516948

[54] Coxeter PD , Del Mar C, Hoffmann TC. Parents’ expectations and experiences of antibiotics for acute respiratory infections in primary care. Ann Fam Med 2017; 15: 149–54. 28289114 10.1370/afm.2040 PMC5348232

[55] Halls A , Van’t Hoff C, Little P et al Qualitative interview study of parents’ perspectives, concerns and experiences of the management of lower respiratory tract infections in children in primary care. BMJ Open 2017; 7: e015701. 10.1136/bmjopen-2016-015701 PMC5640115 28918409

[56] Szymczak JE , Klieger SB, Miller M et al What parents think about the risks and benefits of antibiotics for their child’s acute respiratory tract infection. J Pediatric Infect Dis Soc 2018; 7: 303–9. 28992328 10.1093/jpids/pix073

[57] Van Hecke O , Butler CC, Wang K et al Parents’ perceptions of antibiotic use and antibiotic resistance (PAUSE): a qualitative interview study. J Antimicrob Chemother 2019; 74: 1741–7. 30879040 10.1093/jac/dkz091 PMC6524473

[58] Warembourg M , Lonca N, Filleron A et al Assessment of anti-infective medication adherence in pediatric outpatients. Eur J Pediatr 2020; 179: 1343–51. 32140853 10.1007/s00431-020-03605-8

[59] Diorio C , Tomlinson D, Boydell KM et al Attitudes toward infection prophylaxis in pediatric oncology: a qualitative approach. PLoS One 2012; 7: e47815. 23112849 10.1371/journal.pone.0047815 PMC3480391

[60] Weiss SL , Fitzgerald JC, Pappachan J et al Global epidemiology of pediatric severe sepsis: the sepsis prevalence, outcomes, and therapies study. Am J Respir Crit Care Med 2015; 191: 1147–57. 25734408 10.1164/rccm.201412-2323OC PMC4451622

[61] Hartman ME , Linde-Zwirble WT, Angus DC et al Trends in the epidemiology of pediatric severe sepsis. Pediatr Crit Care Med 2013; 14: 686–93. 23897242 10.1097/PCC.0b013e3182917fad

[62] Blinova E , Lau E, Bitnun A et al Point prevalence survey of antimicrobial utilization in the cardiac and pediatric critical care unit. Pediatr Crit Care Med 2013; 14: e280–8. 23823209 10.1097/PCC.0b013e31828a846d

[63] Hsiao AL , Baker MD. Fever in the new millennium: a review of recent studies of markers of serious bacterial infection in febrile children. Current Opin Pediatr 2005; 17: 56–61. 10.1097/01.mop.0000151781.13635.70 15659965

[64] Milcent K , Faesch S, Gras-Le Guen C et al Use of procalcitonin assays to predict serious bacterial infection in young febrile infants. JAMA Pediatr 2016; 170: 62–9. 26595253 10.1001/jamapediatrics.2015.3210

[65] Nijman RG , Moll HA, Vergouwe Y et al C-reactive protein bedside testing in febrile children lowers length of stay at the emergency department. Pediatr Emerg Care 2015; 31: 633–9. 26181498 10.1097/PEC.0000000000000466

[66] Lautz AJ , Dziorny AC, Denson AR et al Value of procalcitonin measurement for early evidence of severe bacterial infections in the pediatric intensive care unit. J Pediatr 2016; 179: 74–81.e2. 27587074 10.1016/j.jpeds.2016.07.045 PMC5217746

[67] Weiss SL , Peters MJ, Alhazzani W et al Surviving sepsis campaign international guidelines for the management of septic shock and sepsis-associated organ dysfunction in children. Pediatr Crit Care Med 2020; 21: e52–e106. 32032273 10.1097/PCC.0000000000002198

[68] Children’s Hospital Association . Improving Pediatric Sepsis Outcomes (IPSO) is Successfully Challenging Sepsis. https://www.childrenshospitals.org/programs-and-services/quality-improvement-and-measurement/collaboratives/sepsis.

[69] Weiner-Lastinger LM , Abner S, Benin AL et al Antimicrobial-resistant pathogens associated with pediatric healthcare-associated infections: summary of data reported to the National Healthcare Safety Network, 2015–2017. Infect Control Hosp Epidemiol 2020; 41: 19–30. 31762428 10.1017/ice.2019.297 PMC8276251

[70] Foglia EE , Fraser VJ, Elward AM. Effect of nosocomial infections due to antibiotic-resistant organisms on length of stay and mortality in the pediatric intensive care unit. Infect Control Hosp Epidemiol 2007; 28: 299–306. 17326020 10.1086/512628

[71] Same RG , Hsu AJ, Cosgrove SE et al Antibiotic-associated adverse events in hospitalized children. J Pediatric Infect Dis Soc 2021; 28: 622–8. 10.1093/jpids/piaa173 PMC8162628 33452808

[72] Chiotos K , TammaPD, GerberJS. Antibiotic stewardship in the intensive care unit: challenges and opportunities. Infect Control Hosp Epidemiol2019; 40: 693–8.3104685110.1017/ice.2019.74

[73] Darby JM , Linden P, Pasculle W et al Utilization and diagnostic yield of blood cultures in a surgical intensive care unit. Crit Care Med 1997; 25: 989–94. 9201052 10.1097/00003246-199706000-00016

[74] Kiragu AW , Zier J, Cornfield DN. Utility of blood cultures in postoperative pediatric intensive care unit patients. Pediatr Crit Care Med 2009; 10: 364–8. 19325504 10.1097/PCC.0b013e3181a31bb7

[75] Tran CA , Zschaebitz JV, Spaeder MC. Epidemiology of blood culture utilization in a cohort of critically ill children. J Pediatr Intensive Care 2019; 8: 144–7. 31402991 10.1055/s-0038-1676993 PMC6687448

[76] Doern GV , Carroll KC, Diekema DJ et al Practical guidance for clinical microbiology laboratories: a comprehensive update on the problem of blood culture contamination and a discussion of methods for addressing the problem. Clin Microbiol Rev 2019; 33: e00009-19. 31666280 10.1128/CMR.00009-19 PMC6822992

[77] Bates DW , Goldman L, Lee TH. Contaminant blood cultures and resource utilization. The true consequences of false-positive results. JAMA 1991; 265: 365–9. 1984535

[78] Alahmadi YM , Aldeyab MA, McElnay JC et al Clinical and economic impact of contaminated blood cultures within the hospital setting. J Hosp Infect 2011; 77: 233–6. 21216032 10.1016/j.jhin.2010.09.033

[79] Woods-Hill CZ , Fackler J, Nelson McMillan K et al Association of a clinical practice guideline with blood culture use in critically ill children. JAMA Pediatr 2017; 171: 157–64. 27942705 10.1001/jamapediatrics.2016.3153

[80] Woods-Hill CZ , Lee L, Xie A et al Dissemination of a novel framework to improve blood culture use in pediatric critical care. Pediatr Qual Saf 2018; 3: e112. 30584639 10.1097/pq9.0000000000000112 PMC6221585

[81] Woods-Hill CZ , Koontz DW, Voskertchian A et al Consensus recommendations for blood culture use in critically ill children using a modified Delphi approach. Pediatr Crit Care Med 2021; 22: 774–84. 33899804 10.1097/PCC.0000000000002749 PMC8416691

[82] Xie A , Woods-Hill CZ, King AF et al Work system assessment to facilitate the dissemination of a quality improvement program for optimizing blood culture use: a case study using a human factors engineering approach. J Pediatric Infect Dis Soc 2019; 28: 39–45. 10.1093/jpids/pix097 29165616

[83] Woods-Hill CZ , Koontz DW, King AF et al Practices, perceptions, and attitudes in the evaluation of critically ill children for bacteremia: a national survey. Pediatr Crit Care Med 2020; 21: e23–9. 31702704 10.1097/PCC.0000000000002176 PMC6942229

[84] Pittet D , Tarara D, Wenzel RP. Nosocomial bloodstream infection in critically ill patients. Excess length of stay, extra costs, and attributable mortality. JAMA 1994; 271: 1598–601. 8182812 10.1001/jama.271.20.1598

[85] Kelly M , Conway M, Wirth K et al Moving CLABSI prevention beyond the intensive care unit: risk factors in pediatric oncology patients. Infect Control Hosp Epidemiol 2011; 32: 1079–85. 22011534 10.1086/662376 PMC4026036

[86] Simon A , Ammann RA, Bode U et al Healthcare-associated infections in pediatric cancer patients: results of a prospective surveillance study from University Hospitals in Germany and Switzerland. BMC Infect Dis 2008; 8: 70. 18500998 10.1186/1471-2334-8-70 PMC2408583

[87] Morgan JE , Phillips B, Stewart LA et al Quest for certainty regarding early discharge in paediatric low-risk febrile neutropenia: a multicentre qualitative focus group discussion study involving patients, parents and healthcare professionals in the UK. BMJ Open 2018; 14: e020324. 10.1136/bmjopen-2017-020324 PMC5961608 29764879

[88] Morgan JE , Cleminson J, Stewart LA et al Meta-ethnography of experiences of early discharge, with a focus on paediatric febrile neutropenia. Support Care Cancer 2018; 26: 1039–50. 29285558 10.1007/s00520-017-3983-2 PMC5847030

[89] Szymczak JE , Getz KD, Madding R et al Identifying patient- and family-centered outcomes relevant to inpatient versus at-home management of neutropenia in children with acute myeloid leukemia. Pediatr Blood Cancer 2018; 65: e26927. 10.1002/pbc.26927 PMC6857179 29286570

[90] Murni I , MacLaren G, Morrow D et al Perioperative infections in congenital heart disease. Cardiol Young 2017; 27(Suppl 6): S14–21. 29198258 10.1017/S1047951117002578

[91] Herrup EA , Yuerek M, Griffis HM et al Hospital-acquired infection in pediatric subjects with congenital heart disease postcardiotomy supported on extracorporeal membrane oxygenation. Pediatr Crit Care Med 2020; 21: e1020–5. 32590829 10.1097/PCC.0000000000002409

[92] Schwenk HT , Kruger JF, Sacks LD et al Use of prospective audit and feedback to reduce antibiotic exposure in a pediatric cardiac ICU. Pediatr Crit Care Med 2021; 22: e224–32. 33258575 10.1097/PCC.0000000000002608

[93] Bio LL , Kruger JF, Lee BP et al Predictors of antimicrobial stewardship program recommendation disagreement. Infect Control Hosp Epidemiol 2018; 39: 806–13. 29708081 10.1017/ice.2018.85

[94] Porter ME , Lee TH. The strategy that will fix health care. Harv Bus Rev 2013; 91: 50–70.23898735

[95] Morris AM , Calderwood MS, Fridkin SK et al Research needs in antibiotic stewardship. Infect Control Hosp Epidemiol 2019; 40: 1334–43. 31662139 10.1017/ice.2019.276

[96] Bauer MS , Damschroder L, Hagedorn H et al An introduction to implementation science for the non-specialist. BMC Psychol 2015; 3: 32. 26376626 10.1186/s40359-015-0089-9 PMC4573926

[97] Palinkas LA , Aarons GA, Horwitz S et al Mixed method designs in implementation research. Adm Policy Ment Health 2011; 38: 44–53. 20967495 10.1007/s10488-010-0314-z PMC3025112

[98] Barr J , Paulson SS, Kamdar B et al The coming of age of implementation science and research in critical care medicine. Crit Care Med 2021; 49: 1254–75. 34261925 10.1097/CCM.0000000000005131 PMC8549627

[99] King AA , Baumann AA. Sickle cell disease and implementation science: a partnership to accelerate advances. Pediatr Blood Cancer 2017; 64: e26649. 10.1002/pbc.26649 PMC6026013 28556441

[100] Woods-Hill CZ , Papili K, Nelson E et al Harnessing implementation science to optimize harm prevention in critically ill children: a pilot study of bedside nurse CLABSI bundle performance in the pediatric intensive care unit. Am J Infect Control 2021; 49: 345–51. 32818579 10.1016/j.ajic.2020.08.019 PMC7889766

[101] Department of Health and Human Services . https://grants.nih.gov/grants/guide/pa-files/PAR-19-274.html.

[102] Center for Translation Research and Implementation Science . https://www.nhlbi.nih.gov/about/divisions/center-translation-research-and-implementation-science.

[103] Russ AL , Fairbanks RJ, Karsh BT et al The science of human factors: separating fact from fiction. BMJ Qual Saf 2013; 22: 802–8. 10.1136/bmjqs-2012-001450 PMC3786617 23592760

[104] Reason J . Human error: models and management. BMJ 2000; 18: 768–70. 10.1136/bmj.320.7237.768 PMC1117770 10720363

[105] Holden RJ , Carayon P, Gurses AP et al SEIPS 2.0: a human factors framework for studying and improving the work of healthcare professionals and patients. Ergonomics 2013; 56: 1669–86. 24088063 10.1080/00140139.2013.838643 PMC3835697

[106] Bettinger B , Benneyan JC, Mahootchi T. Antibiotic stewardship from a decision-making, behavioral economics, and incentive design perspective. Appl Ergo 2021; 90: 103242. 10.1016/j.apergo.2020.103242 32861088

[107] Djulbegovic B , Hozo I, Beckstead J et al Dual processing model of medical decision-making. BMC Med Inform Decis Mak 2012; 12: 94. 22943520 10.1186/1472-6947-12-94 PMC3471048

[108] Linder JA , Meeker D, Fox CR et al Effects of behavioral interventions on inappropriate antibiotic prescribing in primary care 12 months after stopping interventions. JAMA 2017; 318: 1391–2. 29049577 10.1001/jama.2017.11152 PMC5818848

[109] Rubio DM , Schoenbaum EE, Lee LS et al Defining translational research: implications for training. Acad Med 2010; 85: 470–5. 20182120 10.1097/ACM.0b013e3181ccd618 PMC2829707

[110] Pluye P , Hong QN. Combining the power of stories and the power of numbers: mixed methods research and mixed studies reviews. Annu Rev Public Health 2014; 35: 29–45. 24188053 10.1146/annurev-publhealth-032013-182440

[111] Queirós A , Faria D, Almeida F. Strengths and limitations of qualitative and quantitative research methods. Eur J Educ Studies 2017; 3.

[112] Gubrium JF , Holstein JA. Narrative practice and the transformation of interview subjectivity. In: Gubrium JF, Holstein JA, Marvasti AB et al, eds. The SAGE Handbook of Interview Research. SAGE Publications, 2012; 27–44.

[113] Krueger RA , Casey MA. Focus Groups: a Practical Guide for Applied Research. SAGE Publications, 2014.

[114] Jorgensen D . Participant Observation: a Methodology for Human Studies (Applied Social Research Methods). SAGE Publications, 1989.

[115] Sayood SJ , Venkatram C, Newland JG et al Experiences from the missouri antimicrobial stewardship collaborative: a mixed methods study. Infect Control Hosp Epidemiol 2020; 41: 1455–7. 32746951 10.1017/ice.2020.318 PMC7967295

[116] Gerber JS , Prasad PA, Fiks AG et al Effect of an outpatient antimicrobial stewardship intervention on broad-spectrum antibiotic prescribing by primary care pediatricians: a randomized trial. JAMA 2013; 309: 2345–52. 23757082 10.1001/jama.2013.6287

[117] Lum EP , Page K, Whitty JA et al Antibiotic prescribing in primary healthcare: dominant factors and trade-offs in decision-making. Infect Dis Health 2018; 23: 74–86. 10.1016/j.idh.2017.12.002 38715307

